# ^129^I and its species in the East China Sea: level, distribution, sources and tracing water masses exchange and movement

**DOI:** 10.1038/srep36611

**Published:** 2016-11-16

**Authors:** Dan Liu, Xiaolin Hou, Jinzhou Du, Luyuan Zhang, Weijian Zhou

**Affiliations:** 1 State Key Laboratory of Loess and Quaternary Geology, Institute of Earth Environment, Chinese Cademy of Sciences, Xi’an 710061, China; 2 University of Chinese Academy of Sciences, Beijing 100049, China; 3 Technical University of Denmark, Center for Nuclear Technologies, Risø Campus, Roskilde 4000, Denmark; 4 State Key Laboratory of Estuarine and Coastal Research, East China Normal University, Shanghai, 200062, China

## Abstract

Anthropogenic ^129^I as a long-lived radioisotope of iodine has been considered as an ideal oceanographic tracer due to its high residence time and conservative property in the ocean. Surface water samples collected from the East China Sea (ECS) in August 2013 were analyzed for ^129^I, ^127^I and their inorganic chemical species in the first time. The measured ^129^I/^127^I ratio is 1–3 orders of magnitude higher than the pre-nuclear level, indicating its dominantly anthropogenic sources. Relatively high ^129^I levels were observed in the Yangtze River and its estuary, as well as in the southern Yellow Sea, and ^129^I level in seawater declines towards the ECS shelf. In the open sea, ^129^I and ^127^I in surface water exists mainly as iodate, while in Yangtze River estuary and some locations, iodide is dominated. The results indicate that the Fukushima nuclear accident has no detectable effects in the ECS until August 2013. The obtained results are used for investigation of interaction of various water masses and water circulation in the ECS, as well as the marine environment in this region. Meanwhile this work provides essential data for evaluation of the possible influence of the increasing NPPs along the coast of the ECS in the future.

^129^I (15.7 Ma) is naturally produced by cosmic ray induced spallation of xenon in the atmosphere and fission of uranium on the earth with a ^129^I/^127^I atomic ratio of 1.5 × 10^−12^ in the pre-nuclear seawater samples^1^. While large amount of ^129^I has been released to the environment by human nuclear activities, among them atmospheric nuclear weapons testing (NWT) in 1945–1980 and spent nuclear fuel reprocessing are two major contributions of ^129^I in the environment^2^. The large and well documented ^129^I releases and the high residence time of iodine in the ocean make ^129^I an ideal oceanographic tracer, a number of studies have been carried out in the North Atlantic and Arctic Oceans using ^129^I and its species for water circulation and marine environment investigation^3^,^4^,^5^. A few investigation of ^129^I in the Pacific Ocean were reported^6^,^7^,^8^. However, investigation of ^129^I in the East China Sea (ECS) and the Yellow Sea (YS) is rare. The Fukushima nuclear accident happened in March 2011 has released a large amount of radioactive substance to the atmosphere and seas. The measurement of ^129^I in the Fukushima offshore seawater has shown a significant Fukushima derived ^129^I signals^9^,^10^. However, there was no report showing the Fukushima derived radioactive substance has transported to the ECS and YS. In the recent years, 30 nuclear power reactors have been installed in the coastal region of China. The environmental safety and impact of nuclear facilities are highly concerned. ^129^I, as a fission product, can be used to monitor and evaluate the impact of the nuclear facilities; this requires a baseline value of ^129^I. However, no such ^129^I baseline in seawater along the coastal region of China is available.

Iodine is one of redox sensitive elements; it occurs mainly as iodide (I^−^) and iodate (IO_3_^−^) with minor organic iodine in seawater. Iodate is thermodynamically stable and predominant in the open sea^11^,^12^, but can be converted to iodide in surface photic zones as well as in oxygen-deprived basins^13^. Reduction of IO_3_^−^ to I^−^ in seawater is often related to biological activity such as phytoplankton^14^, microorganism^15^. The speciation analysis of the anthropogenic ^129^I can provide critical information for understanding the marine environment and process.

Due to the increased discharges of pollutants and nutrients, red tide frequently occurs in the ECS and YS in the recent years. Speciation analysis of iodine isotopes can provide a new insight to investigate and evaluate the impact of the pollution. To our best knowledge, no data on the species of iodine isotopes in the ECS has been reported. There are a numbers of water masses and/or water currents in the ECS. The interaction among these water masses directly influences the environment of the ECS. Investigation of the ^129^I distribution in this region will provide an insight into the exchange and interaction of these water masses.

In this work, surface seawater samples collected in the ECS and its adjacent region were analyzed for iodine isotopes and their species, aiming to provide a baseline dataset of the distribution of ^129^I and ^127^I in this region and to trace the exchange and circulation of water masses in this region. The chemical species of ^129^I and ^127^I can be used to investigate the marine circumstance and environmental process in the ECS.

## Results

### Distribution of ^129^I and ^127^I in the ECS

**Figure 1:**
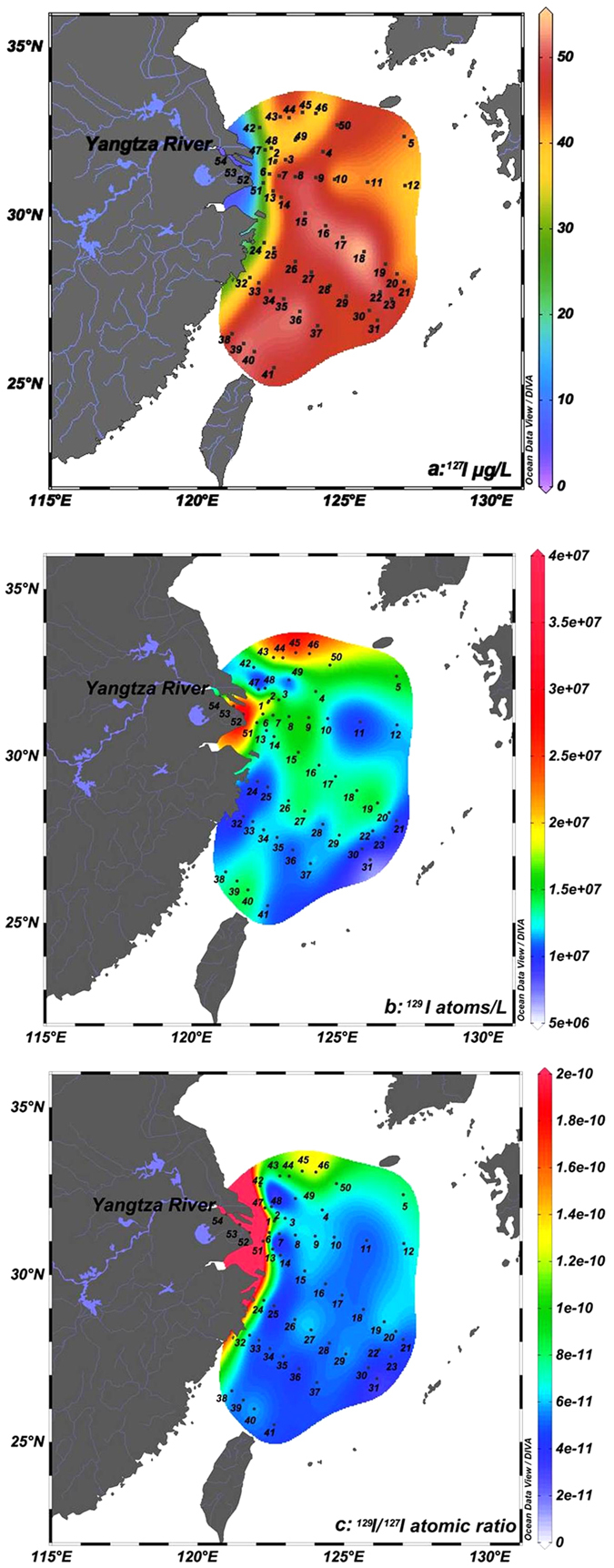
Spatial distribution of ^127^I (**a**) and ^129^I (**b**) concentrations and ^129^I/^127^I atoms ratios (**c**) in the surface water in the ECS. This figure was generated using free software ODV4.7.8 (Schlitzer, R., Ocean Data View, http://odv.awi.de, 2015).

The analytical results of ^129^I and ^127^I in the surface seawater in the ECS are listed in Table S2 (Supplementary information). ^127^I concentrations in the ECS vary significantly in 3.1–54.7 μg/L, the highest value (54.7 μg/L) was observed in the open sea area, which is 8–18 times higher than the lowest values (3.1–7.1 μg/L) in the Yangtze River mouth (station 52–54, Fig. 1a, Supplementary Table S2). In the Yangtze River estuary (stations 24, 32, 42, 47, 51,), the ^127^I concentrations are relative low in 25.0–37.0 μg/L. Enhanced ^127^I concentrations (43.2 μg/L in average) were observed the Yangtze River estuary (station 1, 2, 7) and the offshore seawater (stations 3, 13, 14, 25, 33, 38). Further increased concentrations of ^127^I ranging in 45.1–51.8 μg/L occurred in the southern ECS (stations 26–29, 34–36, 39–41). In the middle and southeast of ECS (stations 15–18, 22), the highest concentrations of ^127^I (50.15–54.74 μg/L) were measured (Fig. 1a, Table S2).

^129^I concentrations vary from 0.7 × 10^7^ to 4.0 × 10^7^ atoms/L (Fig. 1b, Table S2). The high values (1.5–4.0) × 10^7^ atoms/L) were observed in the Yangtze River mouth (stations 52–54) and the Yangtze River estuary (stations 1, 2, 6–9), as well as in the southern Yellow Sea (stations 43–46). The ^129^I concentrations decrease gradually from the Yangtze River estuary towards east and southeast to the ECS shelf. Low ^129^I concentrations (0.8–1.1) × 10^7^ atoms/L were measured in the border of the YS and ECS (stations 47–49). In addition, ^129^I concentrations ((0.7–1.2) × 10^7^ atoms/L) in the eastern ECS (stations of 21, 23, 30, 31) and coastal area in the southern ECS (stations 24, 25, 32) are also low. While, intermediate level ((1.0–1.5) × 10^7^ atoms/L) occurred in the middle and southern ECS (stations of 38–40, 33–37, 26–29 and 13–20) (Fig. 1b, Table S2).

^129^I/^127^I atomic ratios vary significantly from 3.2 × 10^−11^ to 1.2 × 10^−9^ (Fig. 1c, Table S2), with a similar variation trend as the ^129^I concentrations. The highest ^129^I/^127^I atomic ratios of (90–120) × 10^−11^ occurred in the Yangtze River mouth (stations 52–54), which are more than 10 times higher than those in the ECS (<10 × 10^−11^). The lowest ^129^I/^127^I ratios ((3.1–6.4) × 10^−11^) were observed in the eastern and southern ECS (stations 16–20, 21–23, 30–31, 33–37, 39, 41). While, relative high ^129^I/^127^I ratios were measured in the southern YS (stations 42–46) (8.9–13.3) × 10^−11^) and in the Yangtze River estuary (stations 1–3, 6–7, 51) (7.2–13.3) × 10^−11^).

### Chemical species of ^127^I and ^129^I and their distribution

**Figure 2:**
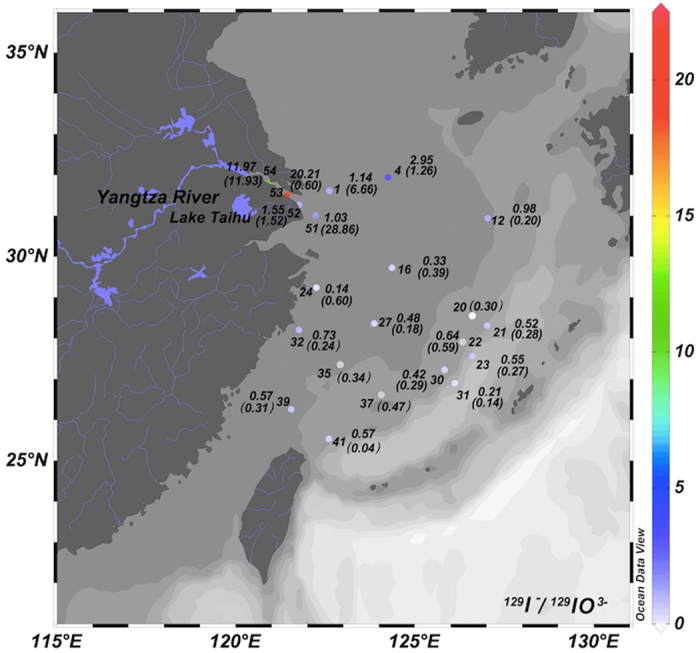
Distribution of iodide/iodate ratios for ^129^I (upper number) and ^127^I (number in parentheses) in seawater in the ECS. This figure was generated using free software ODV4.7.8 (Schlitzer, R., Ocean Data View, http://odv.awi.de, 2015).

Some of surface seawater samples in the ECS were analyzed for ^129^I and ^127^I in iodide (I^−^) and iodate (IO_3_^−^) species (Supplementary Table S3). To better understanding the distribution and variation of iodine species, molar ratios of ^129^I^−^/^129^IO_3_^−^ and ^127^I^−^/^127^IO_3_^−^ are presented in Fig. 2. In general, ^129^I^−^/^129^IO_3_^−^ ratios are higher than ^127^I^−^/^127^IO_3_^−^ in seawater for most sampling stations (Fig. 2). Except for the sampling stations along the Yangtze River mouth (stations 52–54) with ^127^I^−^/^127^IO_3_^−^ ratios of 0.6–11.9, in the Yangtze River estuary (stations 1 and 51) and station 4 with ^127^I^−^/^127^IO_3_^−^ ratios of 1.3–28.9, iodide/iodate molar ratios for both ^129^I and ^127^I were less than 1 in open sea water (Fig. 2 and Supplementary Table S3). The highest ^129^I^−^/^129^IO_3_^−^ ratios (12.0–20.2) were observed in waters at the Yangtze River mouth (stations 53 and 54). While a considerable decrease of ^129^I^−^/^129^IO_3_^−^ ratio (1.0–1.6) were measured in the Yangtze River estuary towards to the ECS at sampling stations 1 and 51. Low ^129^I^−^/^129^IO_3_^−^ molar ratios was observed in waters from the south of the Hangzhou Bay and in the open sea waters further to 30°N in the center of the ECS (stations 16–31) and the southern ECS (stations 27–41).

## Discussion

^129^I concentrations ((0.73–3.99) × 10^7^ atoms/L) and the ^129^I/^127^I atomic ratios ((3.1–120) × 10^−11^) in the surface water in the ECS is 1–3 orders of magnitude higher than the pre-nuclear value (1.5 × 10^−12^ for ^129^I/^127^I ratio and 0.043 × 10^7^ atoms/L for ^129^I concentration)^16^, indicating the dominating anthropogenic source of ^129^I in the surface seawater in the ECS. The highest ^129^I level ((1.2–4.0) × 10^7^ atoms/L for ^129^I concentrations and (10.1–119) × 10^−11^ for ^129^I/^127^I atomic ratios) was observed in Yangtze River mouth and a relative high ^129^I levels of (1.0–2.5) × 10^7^ atoms/L for ^129^I concentrations and (7.0–12) × 10^−11^ for ^129^I/^127^I ratios were measured in the southern Yellow Sea, and the ^129^I levels decline towards to the ECS shelf, the lowest ^129^I levels ((3.1–6.4) × 10^−11^ for ^129^I/^127^I ratios) in the investigated area were measured in the ECS shelf and southern ECS.

The measured ^129^I level in surface water in the ECS is more than one order of magnitude lower than that in contaminated seawater. In the North Sea, Baltic Sea, Norwegian Sea and Arctic, ^129^I/^127^I ratios of 10^−8^–10^−6^ were reported because of huge amount of ^129^I discharged from the European nuclear fuel reprocessing plants (NFRPs)at Sellafield (UK) and La Hague (France) has dispersed with sea current to these area^4^,^17^,^18^,^19^,^20^. In the Fukushima offshore water, the ^129^I/^127^I ratios reached to 2.2 × 10^−9^ (6.3 × 10^8^ atoms/L for ^129^I concentration) due to the releases from the Fukushima accident^10^ (Supplementary Table S4).

**Figure 3:**
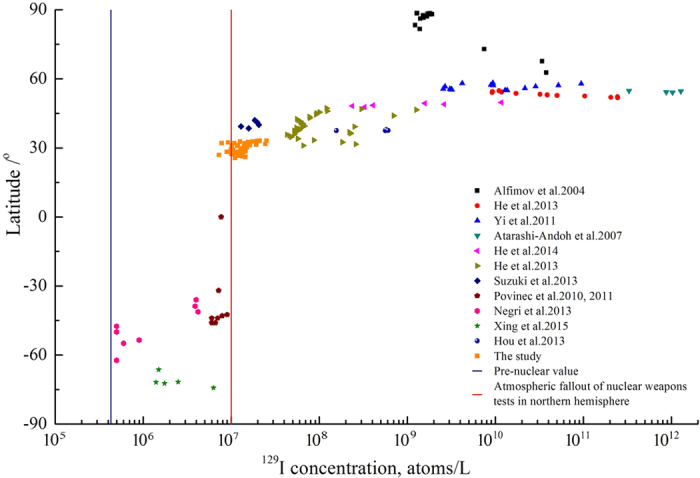
Comparison of ^129^I concentrations in surface seawater from the ECS and other locations.

Compared to the region with similar latitude as the ECS but no direct contamination by human nuclear activity, the ^129^I level in the ECS is comparable to those observed in adjacent sea around Japan before Fukushima accident, where ^129^I concentrations of (1.2–2.1) × 10^7^ atoms/L (with ^129^I/^127^I ratios of 4.64–7.2 × 10^−11^) were report^6^. But it is lower than those in the north Atlantic (31–50°N) (^129^I concentrations of (4.0–12.7) × 10^7^ atoms/L and ^129^I/^127^I ratios of (2–57) × 10^−10^)^3^. The higher ^129^I level in the North Atlantic results from the leakage and southwards dispersion of the high reprocessing derived ^129^I water in the English Channel. In addition, the ^129^I in the ECS is also comparable to those observed in the Indian Ocean (40–50°S), where the ^129^I concentrations were (0.60–1.52) × 10^7^ atoms/L (Fig. 3)^21^,^22^. Based on above, it can conclude that ^129^I in the ECS water fall in normal global fallout background level.

However, the ^129^I in the ECS continental shelf are higher than that in the South Atlantic Ocean and the Antarctic ((0.05–0.4) × 10^7^ atoms/L for ^129^I concentrations and (0.28–2.9) × 10^−11^ for ^129^I/^127^I ratios) (Fig. 3, Supplementary Table S4)^23^,^24^. This should be attributed to less fallout of NWTs derived radioactive substances in the south hemisphere.

The dominantly anthropogenic ^129^I in the ECS might originate from 1) releases from nuclear facilities in coastal region of the ECS; 2) fallout of the atmospheric NWTs in 1945–1980; 3) releases from NFRPs and long distance transport; 4) nuclear accidents (e.g. Chernobyl and Fukushima accidents).

Although huge amount of ^129^I has been produced during the operation of the nuclear power plants (NPPs)^1^, most of it was kept in the fuel elements and only very tiny fraction might be released if leakage of fuel elements occurred. Up to 2013, total 13 nuclear power reactors in 4 NPPs including Qianshan (30.43°N, 120.92°E), Fangjiashan (30.43°N, 120.92°E), Sanmen (29.04°N, 121.62°E), and Fuqing (25.53°N, 119.52°E) in coastal region in the ECS were in operation. However, there is no report on the significant releases of fission products from any of these NPPs. An investigation on the ^129^I in the seawater surround a Chinese NPP in the coastal region of the ECS has confirmed no excessive ^129^I in the seawater in 10 km range of this NPP^25^. There is no any NFRPs or other nuclear facilities surrounding the ECS. Therefore, the direct contribution of nuclear facilities to the ^129^I in the ECS is negligible.

It has been estimated that 50–150 kg of ^129^I has been released to the atmosphere from the atmospheric NWTs in 1945–1980^26^. All NWT sites are far from the ECS and the catchment of rivers flowing into the ECS. The nearest testing site at Lop Nor (41°30′N, 88°30′E) is about 3000 km northwest of the ECS, where only 23 atmospheric NWTs were implemented in 1964–1980, An investigation of a sediment core collected in Jiaozhou Bay in the YS has observed a small but obvious ^129^I signal in 1972–1976, which was attributed to the contribution of Chinese NWTs in this period^27^. Considering the very small ^129^I signal of Chinese NWTs in the corresponding layer of this sediment core and these tests were implemented more than 30 years ago, the direct contribution of the Chinese NWTs to the ^129^I in the ECS, especially the present surface seawater, should be insignificant. However, the global fallout of ^129^I from all atmospheric NWTs might be one of ^129^I sources in the ECS because of the large releases. A relative high ^129^I signal of the global fallout peaked in 1962–1963 has been observed in the sediment core collected in the Jiaozhou Bay^27^. Compared to the direct deposition of ^129^I in the ECS in early time of atmospheric NWTs, the leaching of ^129^I from the earth surface by rainwater and subsequent transport to the ECS through rivers might be one of important source and pathway of ^129^I in the ECS.

105 NWTs have been conducted in the Pacific Proving Grounds (PPG) in the Marshall Islands (11°N) in 1946–1962, where is about 5000 km southeast of the ECS. Besides those injected to the stratosphere and contributed to the global fallout, large amount of radioactive substances from these NWTs were deposited in the southern Pacific Ocean surrounding the Marshall Islands (close-in fallout). A number of investigations have reported that the close-in fallout of plutonium in the PPG has being carried by the north equatorial current, which is followed by the Kuroshio Current (KC) and continuously transported to the South China Sea and ECS^28^,^29^. The constantly significant PPG derived Pu signal observed in the ECS was attributed to the continuous re-suspension of the highly deposited plutonium in the sediment in the PPG region^28^,^29^. Compared to plutonium, iodine is water soluble and conservative in ocean, the close-in fallout of ^129^I at the PPG can be also transported to the ECS in early time of the NWTs at PPG. The ^129^I in the sediment core collected in Jiaozhou Bay in the YS has recorded a small but obvious PPG derived signal of ^129^I in 1957^27^. Meanwhile, a similar ^129^I peak occurred in 1957–1958 in coral samples collected in the coastal region in Philippine has also been reported^30^, which suggests that PPG derived ^129^I might have a contribution to ^129^I in the ECS. However, the distribution of ^129^I in the ECS shows the lowest ^129^I level in the east edge of the ECS, i.e. the KC belt, indicating no significant amount of ^129^I in the ECS was transported from the PPG through the KC. This might be attributed that the NWTs at PPG has terminated in 1962, and transit time from PPG to the ECS is less than 2 years, the high water soluble ^129^I released from these NWTs did not significantly deposited and remained in the sediment in the PPG area, instead it was quickly diluted and dispersed to a large area with sea currents. Therefore, unlike plutonium, the contribution of the PPG to the ^129^I in the present surface water in the ECS is insignificant.

Releases from the NFRPs are so far the largest source of ^129^I in the environment^1^,^2^,^3^,^4^. A NFRP at Tokai, Japan is the nearest NFRP to the ECS (about 1500 km northeast), which was operated in 1977–2007. A constant low ^129^I level observed in the precipitation collected in 1979–2003 in Ishigaki-shima (24°N, 124°E)^31^ located in the east edge of the ECS area indicated no significant influence of Tokai NFRP to ^129^I level in the ECS. The NFRP in northwest China is far away from the ECS (>5000 km) and the catchment of the rivers (>2000 km) flowing into the ECS. ^129^I in the sediment core collected in the YS and surface soil collected in southern China did not show any contribution of Chinese NFRP. Therefore, there is no significant contribution of Chinese NFRP to ^129^I in the ECS.

Besides atmospheric releases, the two NFRPs at Sellafield (UK) and La Hague (France) have discharged more than 6500 kg of ^129^I to the seas, which was dispersed to a large area in northern North Atlantic Ocean by sea currents^26^, and an annual discharge of ^129^I still remains at a high level of about 250 kg/y. Re-emission of the reprocessing derived ^129^I from the contaminated seawater to the atmosphere has become a major source of ^129^I in the air in recent years^32^. The ^129^I record in the sediment core from the YS has demonstrated that the atmospheric releases of the European NFRPs and re-emission of the reprocessing derived ^129^I in the sea is a major source of ^129^I in the present sediment in the YC^27^. ^129^I in the Japan Sea was also attributed to the European NFRPs^6^. All these suggest that the European NFRPs might be the major source of ^129^I in the ECS besides the global fallout of the NWTs.

The significantly higher ^129^I level observed in Yangtze River mouth compared to the stations in the ECS indicates that riverine input might be an important source of ^129^I in the ECS. Yangtze River is the longest river (6300 km length) in China with a catchment area of 1,808,500 km^2^ located in southern China (25–34°N). The fallout of ^129^I on the soil of the catchment has being continuously leached by rain and entered to river water, causing a high ^129^I level in the Yangtze River. The high level ^129^I in the southern YS indicate a higher deposited and riverine input of ^129^I from the Yellow River. The YS and the catchment area of the Yellow River are located in north China (32.5–40°N), and therefore received higher fallout of ^129^I compared to the ECS, because most of NWT sites and NFRPs are located in the middle and higher latitude in the Hemisphere^16^,^33^. ^129^I levels in soil and seaweed in the north China and the YS is higher than that in the south China and the ECS^34^,^35^.

Besides the Yangtze River, there are more than 40 rivers flowing into the ECS, among them the relative big ones include Qiantang River (55,600 km^2^ catchment area and 688 km length), Min River (61,000 km^2^ catchment area and 577 km length) and Ou River (18,000 km^2^ catchment and 388 km length). Considering total surface area of 1,940,000 km^2^, the riverine input from the terrestrial deposition is one of major contribution of ^129^I in the ECS. This is also supported by investigation on the distributions of another important fallout radionuclide ^137^Cs in the sediment in the Yangtze River estuary, which showed that the major part of ^137^Cs in the estuary originated from riverine transport and input^36^,^37^.

It has been estimated that 1.3–6 kg ^129^I has been released from the Chernobyl accident. ^131^I signal in the atmospheric samples collected at northern China in May 1986 immediately after the Chernobyl accident has been observed, and a small signal of Chernobyl accident derived ^129^I has been also observed in the sediment collected in the YS^27^. However, no ^131^I in the atmosphere in southern China was observed in 1986 after the Chernobyl accident. Considering the small releases, far distance, and happened more than 25 years ago, Chernobyl accident contributed insignificant amount of ^129^I in the surface water in the ECS.

Fukushima nuclear accident happened in March 2011 has released 1.2 kg ^129^I including about 0.35 kg ^129^I discharged directly to the sea, which caused an elevated ^129^I level in the Fukushima offshore seawater with the highest ^129^I/^127^I atomic ratio of 2.2 × 10^−9^ in the surface seawater near the Fukushima Dai-ichi NPP^10^. Due to the dominant westerly wind after the accident, the deposition of Fukushima derived radioactive substances were relative low in China, especially in the south China. Consequently, the direct deposition of Fukushima derived ^129^I in the ECS and the catchment of the rivers flowing into the ECS is insignificant. Marine ^129^I from the Fukushima accident was mainly transport eastwards in the North Pacific Ocean with the KC. An elevated ^129^I in the surface seawater has been observed in the North Pacific Ocean in 2013^38^. It is therefore concluded that the Fukushima accident contributed insignificant amount of ^129^I to the ECS.

Based on the marine dispersion model, it was estimated that the Fukushima derived radioactive substances will arrive in the ECS in 2013 and reach to peak value in 2018^39^, however, the relative low ^129^I level in the center and eastern ECS compared to the northern and western ECS indicates that Fukushima accident derived ^129^I has not yet arrived to the ECS, at least not measurable until summer 2013.

The increasing gradient of ^127^I concentrations and declined ^129^I level in the surface water from the Yangtze River estuary towards the ECS shelf (Fig. 1) shows an interaction of Yangtze River water with seawater from the KC and TWC, and the mixing model of these water masses (Fig. 1). The high ^129^I but low ^127^I level in the riverine water and high ^127^I but low ^129^I level in seawater from the TWC and KC makes ^129^I/^127^I atomic ratio a perfect indicator to trace the water masses interaction in this region. From the distribution of ^127^I and ^129^I concentrations and ^129^I/^127^I ratios in the ECS (Fig. 1), it can be observed that the Yangtze River water splits into three branches when it injects into the ECS: one branch moves southwards along the continental shelf of the ECS, extends as far as down to 26°N, showed by rapidly decreased ^129^I concentrations and ^129^I/^127^I ratios along this transport pathway; the second branch moves southeastwards, from the Yangtze River estuary to the continental shelf of the ECS, showed by the decreased ^129^I/^127^I ratios from 1.17 × 10^−9^ to 4.14 × 10^−11^ at 126°E; in the third branch, Yangtze River water moves northeastwards, encountered the cold water mass from Yellow Sea, the cyclone circulation produced by the cold water mass turns this branch to the east and extend to the Jeju Island. This observation in 2013 is supported by an investigation of depth profiles of conductivity, temperature, and drifter trajectory in the ECS water in the summers of 1997 and 1998, showing the Yangtze River water plume moved eastwards to the offshore and further to Jeju Island^40^.

A significantly declined ^129^I level from the southern YS to the ECS was observed for both coast water and offshore water. This trend indicates that the high ^129^I Yellow Sea water moves from north to south, and meet the high ^129^I level Yangtze River water from the Yangtze River estuary in the border of the Yellow Sea and ECS, and then turn to northeast at 32°N. ^129^I concentrations in the surface seawater at the border of the ECS and YC are 1–3 times lower than that in the surrounding region. Meanwhile the ^127^I concentrations in this region (sampling stations 47 and 48) are relative low (30–33 μg/L). ^129^I absent ground seepage water input along the shore might occur here. A hypoxic condition observed at sampling station 48 (Supplementary Table S1) supports this assumption. Low salinity and low temperature observed at sampling stations 47–49 also support this assumption. An upwelling zone in the region of 122°00′–123°30′E, 31°–32°30′N (stations 47–48) has also been observed in summer 1985^41^. Therefore, the low ^129^I and ^127^I level in this region might results from the groundwater seepage and upwelling of seawater in this region.

Low ^129^I region observed at the continental slope of the ECS (stations 28, 36 and 41) might indicate a branch of the KC in the ECS in this region. The KC formed from the subtropical mode water of the central North Pacific^42^ contains low ^129^I (Fig. 1). The distribution of ^129^I level indicates that the major KC flows northeastwards along the out edge of the ECS continental shelf, and a small branch moves northwards when it enters the ECS along a transection at stations 28, 36 and 41. The low ^129^I level at stations 11 and 12 indicates that another branch of the KC moves northwestwards and mixed with the Yangtze River water and the Taiwan warm current (TWC) at about 31°N in the northern ECS.

Low ^129^I level was also observed along the south coast of the ECS at stations 24, 25 and 32. This might indicate that the high ^129^I level Yangtze River water does not move southerly along the coast. Considering ^127^I concentrations are also lower in this region and there is not big river flowing into the ECS in this region, it can be assumed that the groundwater seepage in this region might also occur.

^127^I mainly exists as iodate with iodide/iodate molar ratios of 0.04–0.60 in most sampling stations in the ECS, this is similar to those observed in the North Sea (0.11–0.48)^4^,^43^. The lowest ^127^I^−^/^127^IO_3_^−^ molar ratio of 0.04 was observed at sampling station 41, close to the Mien Hwa Canyon (MHC) with a northwestwards KC intrusion branch. This might be attributed to the Kuroshio subsurface water (KSW) upwelling in this region^44^,^45^, because iodate is normally dominated in deep seawater due to less biological activity. The low iodide/iodate ratios in the continental shelf edge of the ECS might also result from some vortex upwelling, when the KC moves northeastwards along the edge of continental shelf^45^. Besides the upwelling water, the low iodide fraction (or oxidation condition) in the KC water might be another reason. No significant correlation between ^127^I^−^/^127^IO_3_^−^ and ^129^I^−^/^129^IO_3_^−^ ratios (R^2^ = 0.007) was observed for all seawater samples investigated. Meanwhile ^129^I^−^/^129^IO_3_^−^ ratios are normally higher than ^127^I^−^/^127^IO_3_^−^ ratios. This is attributed to the different sources of ^127^I and ^129^I in these waters. ^129^I in the ECS mainly originates from the atmospheric fallout and riverine input, whereas ^127^I originates from the sea currents which flow into the ECS.

In the Yangtze River mouth, both of ^129^I and ^127^I originated from the rainwater leaching of soil in the catchment, the similar high iodide/iodate ratios of 12 for both ^127^I and ^129^I were measured at station 54. This indicates iodide is the dominant species of iodine isotopes. This is similar as that observed in the Elbe River in the North Sea^4^, indicating that the Yangtze River water, at least in the mouth area, is reductive/anoxic water. Declined ^127^I^−^/^127^IO_3_^−^ ratios of 0.6–1.5 were measured in the downstream water at stations 52 and 53. This might be attributed to the tidal action and sea water intrusion showed by an elevated salinity (0.5) at sampling station 52. The similar declined ^129^I^−^/^129^IO_3_^−^ ratio (1.5) was measured at station 52, whereas a much higher value up to 20 at sampling station 53. Compared to ^127^I, ^129^I^−^/^129^IO_3_^−^ ratios might reflect the water circumstance in this region, because the contribution from the seawater intrusion is minor. The high ^129^I^−^/^129^IO_3_^−^ ratio at sampling station 53 indicates the reductive circumstance of the Yangtze River water remained at this station. The declined ^129^I^−^/^129^IO_3_^−^ ratio at station 52 close to the Yangtze River estuary indicates the effluence of the seawater, and the reductive circumstance is changed, causing oxidation of ^129^I^−^ to ^129^IO_3_^−^.

High ^127^I^−^/^127^IO_3_^−^ ratios (7–29) were measured at sampling stations 1 and 51 in the ECS, which are even higher than that in the Yangtze River mouth. It has been reported that a red tide (phytoplankton blooming) occurred in the estuary of the Yangtze River in August 2013, and the measured concentrations of chlorophyll were 5.6–37.7 mg/m^3^ 46. The red tide phenomenon was also observed at sampling stations 1, 2 and 51 during the sampling expedition in August 2013. The dominant iodide specie in this region might result from the reductive condition due to the phytoplankton blooming in this region^14^,^47^.

High iodide/iodate ratios were also observed at sampling station 4 (1.3 for ^127^I and 2.9 for ^129^I), which are comparable or even higher than that in the Yangtze River mouth (stations 52). An oxygen depleted condition was measured in the water at sampling station 3, 4 and 48 during sampling campaign in August 2013. The dominant iodide specie in this region (sampling sate 3–4) might results from the anoxic condition in this region, which reduce iodate to iodide.

Since the total ^129^I concentrations in the water at the sampling stations 1–4 are similar to those in the southern region at sampling stations 6–9, the elevated ^129^I^−^/^129^IO_3_^−^ ratios in sampling station 3–4 should be attributed to the reduction of ^129^IO_3_^−^ in the sampling region (station 3–4) which was transported from the Yangtze River estuary northwards through the sampling station 6–9. This also indicates that the reduction of iodate in the anoxic region or biologically active region, especially phytoplankton blooming region, is a relative fast process.

## Materials and Methods

### Investigation area

**Figure 4:**
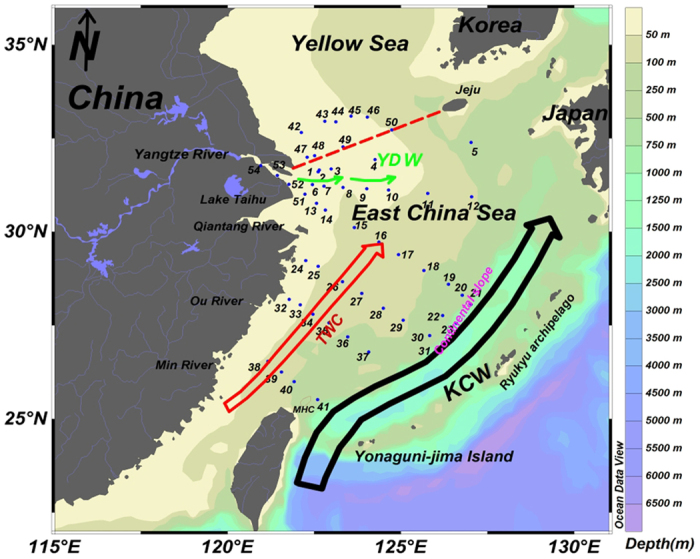
Sampling sites and water masses in the Yangtze River estuary and adjacent seas. Blue dotes represent the sampling sites of surface water, the numbers aside dots are the sample/station code. The red dotted line represents border of the ECS and Yellow Sea. Yangtz River Diluted Water (YDW, summer), Taiwan Warm Current (TWC), Kuroshio Current Water (KCW). This figure was generated using free software ODV 4.7.8 (Schlitzer, R., Ocean Data View, http://odv.awi.de, 2015).

The ECS is the largest marginal sea in the northwestern Pacific, bounded by China, Japan, Ryukyu archipelago, and Korea (Fig. 4). It receives fresh water input from rivers, among them Yangtze River contributes 90% of fresh water input. The KC flows into the ECS through the Channel between Taiwan and Yonaguni-jima Island, and moves northward along the edge of continental shelf. The KC follows the North Equatorial Current in the Pacific Ocean which passes through the PPG in Marshall Islands where 105 NWTs were conducted (11°N) in 1946–1962. The TWC formed by the mixing of the KC surface water and the Taiwan Strait Water, enter the ECS from the Taiwan Strait. In the northern part of the ECS shelf, the Yellow Sea Surface Current flows southwards and participates in the structure of the ECS water masses.

### Collection of the seawater samples

Surface seawater samples (<2 m depth) were collected during the R/V “Dongfanghong 2” cruise in August 2013 using a built-in seawater sampling system in the laboratory of the vessel at 54 sampling stations in the ECS and its adjacent region (Fig. 4, Supplementary Table S1). The temperature and salinity of the seawater samples were measured on-line during sampling (Supplementary Table S-1). Samples were filtered through Φ0.45 μm filter membrane to remove suspended particles *in situ* and stored in 2.5 L polyethylene plastic containers in dark under normal laboratory conditions until analysis.

### Separation of ^127^I and ^129^I species from seawater

A modified procedure from Hou *et al.*^4^ which is based on anion exchange chromatography was applied to separate iodide and iodate from seawater for measurement of ^127^I^−^ and ^127^IO_3_^−^. The detailed procedure is presented in the Supporting information.

A modified co-precipitation method from Luo *et al.*^48^ was used for separation of iodide and total inorganic iodine from seawater for measurement of ^129^I. For separation of iodide, after addition of 0.20 mg of ^127^I^−^ carrier and 200 Bq^125^I^−^ tracer to 1200 ml seawater, NaHSO_3_ solution was added to final concentration of 0.3 mmol/L, and then HCl was added to adjust pH to 5.1, 50 mg Ag^+^ (as 0.10 mmol/L AgNO_3_) was slowly added to the sample under stirring to coprecipitate iodide as AgI-AgCl. For total inorganic iodine, 500 ml seawater was taken to a beaker, 0.20 mg of ^127^I^−^ carrier, 200 Bq ^125^I^−^ (NaI) and 2.0 mmol NaHSO_3_ solution were added. HCl was then added to adjust pH < 2, iodate was reduced to iodide in this condition. 150 mg Ag^+^ was slowly added to the solution under stirring to coprecipitate the formed iodide as AgI-AgCl. The precipitate was washing with 1–7.5% ammonium to obtain a final precipitate of 1–3 mg for AMS measurement of ^129^I.

Procedure blanks for iodide and total inorganic iodine were prepared using the same procedure as for the samples. In this case, deionized water was used instead of the samples.

### Measurement of ^127^I using ICP-MS and ^129^I by AMS

Iodine in the separated iodate and iodide fractions and original seawater was measured used ICP-MS (Thermo Scientific, X series II, USA) after 10 times dilution using 1% ammonium solution. The detection limit of 0.02 ng/mL for ^127^I was obtained.

The separated AgI–AgCl coprecipitate was dried in an oven at 60–70 °C, the dried precipitate was ground to fine powder and mixed with niobium powder (325-mesh, Alfa Aesar, Ward Hill, MA) in a mass ratio of 1:5, which was finally pressed into copper holder. The ^129^I/^127^I atomic ratios in the prepared targets were measured by AMS using 3MV Tandem AMS system (HVEE) in the Xi’an AMS center. ^127^I^5+^ was measured as charges (current) using a Faraday cup and ^129^I^5+^ was measured using a gas ionization detector. All samples were measured for 6 cycles and 5 min per sample in each cycle. The procedure background of ^129^I/^127^I ratio was measured to be 1.0 × 10^−13^. For the samples with ^129^I/^127^I ratio of 10^−10^, the measurement uncertainty is less than 1.75%^49^. A detailed description of AMS system and measurement of ^129^I has been reported elsewhere^50^.

## Additional Information

**How to cite this article**: Liu, D. *et al.*^129^I and its species in the East China Sea: level, distribution, sources and tracing water masses exchange and movement. *Sci. Rep.* **6**, 36611; doi: 10.1038/srep36611 (2016).

**Publisher’s note:** Springer Nature remains neutral with regard to jurisdictional claims in published maps and institutional affiliations.

## Supplementary Material

Supplementary Information
