# Supplementary material for: 129I and its species in the East China Sea: level, distribution, sources and tracing water masses exchange and movement
Source: Sci Rep. 2016 Nov 16;6:36611. doi: 10.1038/srep36611 (PMC5111073; doi:10.1038/srep36611)
Supplement: Supplementary Information [file srep36611-s1.doc]

**Supplementary Information**

**^129^I and its species in the East China Sea: level, distribution, sources and tracing water masses exchange and movement**

Dan Liu^1, 4^, Xiaolin Hou^1, 3,*^, Jinzhou Du^2^, Luyuan Zhang^1^ & Weijian Zhou^1^

**Affiliations**

**­­**^1^ State Key Laboratory of Loess and Quaternary Geology, Institute of Earth Environment, Chinese Cademy of Sciences, Xi’an 710061, China.

^2^ State Key Laboratory of Estuarine and Coastal Research, East China Normal University, Shanghai, 200062, China.

^3^ Technical University of Denmark, Center for Nuclear Technologies, Risø Campus, Roskilde 4000, Denmark.

^4^ University of Chinese Academy of Sciences, Beijing 100049, China.

Correspondence and requests for materials should be addressed to X.H. (email:xiho@dtu.dk, houxl@ieecas.cn,)

***Investigation area***

The ECS is the largest marginal sea in the northwestern Pacific, bounded by China, Japan, Ryukyu archipelago, Korea and Taiwan. It has a broad continental shelf, which plays a vital role in exchanging and transporting material from the continent to the northwestern Pacific Ocean. It receives riverine inputs from the Yangtze River which contributes about 90% fresh water to the ECS every year. The total length of Yangtze River is 6397kmand the total area of its basin is 1,808,500km^2^, which accounts for about 1/5 the total area of China, with 3219 km length from west to eat and 966 km width from north to south. The annual mean discharge is about 28,400 m^3^/s and the mean discharge peaks in August with about 48,000 m^3^/s. [**^1^**](#_ENREF_36)The discharge from the Yangtze River mixes with saline ambient water and forms a water body called the Yangtze River diluted water (YDW).In summer, YDW shows a bimodal structure, including a southward coastal jet and a northeastward spread. [**^2^**](#_ENREF_37) In the eastern ECS**,** the Kuroshio Current (KC) from the south flows northwards by the East Taiwan along the way between Taiwan and Yonaguni-jima Island into the ECS. It moves northward along the edge of continental shelf, and then separates from the shelf and bends east-southeastward and eventually flows into the Pacific Ocean through the Tokara Strait. [**^3^**](#_ENREF_38)**,**[**^4^**](#_ENREF_39)One part of the flow on the left-hand side of the KC separates to become the Tsushima Current. The Kuroshio Current Surface Water (KCSW) is warm and saline, flowing northeastward along the eastern margin of the ECS continental shelf, being dominated by the subtropical mode water of the central North Pacific.The ECS circulation pattern is mainly regulated by the KC, which influence the exchange of water, heat and nutrient between the ECS and the open sea in the ECS shelf, except YDW and KC, there are some water masses which interact with each other, such as the Zhejiang-Fujian Coast Current(ZFCC).It is one part of the ECS Coastal Current and from the Yangtze River estuary, which mainly originate from the runoff of the Yangtze River and the Qiantang River and other rivers into the sea and the mixture of water. It is mainly distributed in the Yangtze River estuary and the south of Zhejiang and Fujian coast. The Taiwan Warm Current (TWC),paralleled with ZFCC, with its upper water formed by the mixing of the KCSW and the Taiwan Strait Water, and its deep water originating from the Kuroshio Current Subsurface Water at the east of Taiwan. Moreover, in the northern part of the ECS shelf, the Yellow Sea Surface Current (YSSC) also participates in the structure of ECS water masses.The ECS shelf water is likely to turn over approximately each two years because of the circulation of currents and the mixing of water masses, which causes considerably rapid exchange of chemical substance between this marginal sea and open ocean.

***Chemical reagents and standards***

All chemical reagents used were of analytical grade and all solutions were prepared using deionized water (18.2 MΩ cm^−1^), ^129^I standard (NIST-SRM-4949c) was purchased from National Institute of Standards and technology (Gaithersburg, USA). Iodide carrier (^127^I^-^) as NaI was purchased from Chaoshan Specialty Chemicals (Guangzhou, China), ^129^I/^127^I ratio in this reagent was measured to be <2×10^−13^. The ^127^I carrier (both iodide and iodate) was prepared using iodine crystal provided by Woodward Corporation (MICAL Specialty Chemicals, New Jersey) with a measured ^129^I/^127^I atomic ratio <2× 10^14^). Bio-Rad AG1-×4 anion exchange resin (50-100 mesh, Cl form, Bio-Rad laboratories, Richmond, CA, USA) were used for separation of iodine species from seawater.

***Methods for separation of ^127^I species from seawater***

In brief, the Bio-Rad AG1×-4 strongly basic anion exchange resin (50-100 mesh) was converted to NO_3_^–^ form and packed in a column (1.0 cm in diameter and 10 cm in height). About 30 ml of seawater was loaded onto the column at a flow rate of 1 ml^.^min^−1^. The column was washed with 15ml deionized water and 15ml 0.2M KNO_3_ solution. The effluent and the rinse solution were combined for the determination of iodate. The iodide on the column was eluted using 15 ml NaClO (10%) solution and 15mldeionized water. The separated samples as well as original seawater samples were diluted by a factor of 20 using 1% NH_3_ for iodate fraction and original seawater and H_2_O for iodide fraction. Iodine in the diluted samples was measured used ICP-MC (Thermo Scientific, X series II, USA) to determine concentration of total ^127^I,^127^I^-^ and ^127^IO_3_^-^. The procedure blanks were also prepared using the same procedure and measured using ICP-MS. The detection limit of 0.02 ng/mL for ^127^I was obtained. Iodide concentration was corrected for chemical yield during column separation.

***Separation of iodide and total inorganic iodine from seawater by co-precipitation for AMS measurement of ^129^ species.***1200 ml of seawater was transferred to a beaker, 0.2 mg ^127^I iodide carrier (NaI), and 200 Bq^125^I^−^were spiked. After addition of NaHSO_3_to 0.3 mmol/l, HNO_3_ (0.5 mol/L) was slowly added under stirring to carefully adjust to pH 5. AgNO_3_ (150 mg Ag^+^) was slowly added under stirring to coprecipitate iodide as AgI. After the mixture was stirred for 1 h, the precipitate was separated by centrifuge. The precipitate was sequentially rinsed with HNO_3_, H_2_O, and 7.5%, 2.5%, and 1% NH_3_·H_2_O to remove Ag_2_SO_3_ and excessive AgCl and AgBr until 1-3 mg of precipitate was obtained. To evaluate the separation efficiency and cross contamination of iodate into iodide fraction,^131^IO_3_^−^and^125^I^-^ tracers were spiked to seawater, iodide was separated as AgI using the above procedure. ^131^I and ^125^I in the AgI precipitate was measured using gamma spectrometry, and the cross-over contamination of iodate in the iodide was calculated by comparing with the total ^131^IO_3_^-^and ^125^I^-^spiked to the sample. It is worth to mention that no ^131^I tracer was used in the analysis of ^129^I and its species in the seawater because of relative high ^129^I contamination in the ^131^I tracers.

**Supplementary TableS1.** Sampling information of the surface water from the East China Sea region

| **Sampling station** | **Date**  **y/m/d** | **Longitude(E)** | **Latitude(N)** | **Temperature**  **°C** | **Salinity**  **/PSU** | **Remarks** |
| --- | --- | --- | --- | --- | --- | --- |
| 1 | 2013.8.7 | 122°36.792′ | 31°36.792′ | 26.91 | 18.77 |  |
| 2 | 2013.8.6 | 122°38.485′ | 31°39.542′ | 25.25 | 28.70 | bloom |
| 3 | 2013.8.7 | 122°58.751′ | 31°41.575′ | 26.33 | 28.02 | hypoxic |
| 4 | 2013.8.7 | 124°15.297′ | 31°56.498′ | 27.87 | 30.33 | hypoxic |
| 5 | 2013.8.10 | 127°00.911′ | 32°23.927′ | 30.05 | 35.54 |  |
| 6 | 2013.8.6 | 122°46.987′ | 31°13.709′ | 25.66 | 27.52 |  |
| 7 | 2013.8.7 | 122°26.209′ | 31°16.409′ | 23.14 | 29.51 |  |
| 8 | 2013.8.12 | 123°19.736′ | 31°11.219′ | 23.38 | 31.12 |  |
| 9 | 2013.8.12 | 124°00.587′ | 31°09.338′ | 26.37 | 30.78 |  |
| 10 | 2013.8.11 | 124°39.443′ | 31°07.536′ | 29.31 | 30.84 |  |
| 11 | 2013.8.11 | 125°46.131′ | 31°01.735′ | 29.91 | 32.77 |  |
| 12 | 2013.8.11 | 127°02.241′ | 30°56.613′ | 29.57 | 31.80 |  |
| 13 | 2013.8.15 | 122°33.841′ | 30°45.993′ | 24.27 | 30.29 |  |
| 14 | 2013.8.15 | 122°49.374′ | 30°35.565′ | 25.82 | 31.16 |  |
| 15 | 2013.8.16 | 123°39.138′ | 30°07.442 | 28.71 | 33.71 |  |
| 16 | 2013.8.16 | 124°21.575′ | 29°43.783′ | 28.97 | 33.64 |  |
| 17 | 2013.8.16 | 124°55.988′ | 29°23.796′ | 30.15 | 33.31 |  |
| 18 | 2013.8.16 | 125°39.338′ | 28°57.931′ | 30.35 | 33.59 |  |
| 19 | 2013.8.17 | 126°22.100′ | 28°36.152′ | 24.19 | 34.66 |  |
| 20 | 2013.8.17 | 126°46.403′ | 28°18.804′ | 25.22 | 34.38 |  |
| 21 | 2013.8.17 | 127°01.134′ | 28°04.351′ | 30.31 | 34.09 |  |
| 22 | 2013.8.23 | 126°12.021′ | 27°46.064′ | 28.38 | 34.09 |  |
| 23 | 2013.8.19 | 126°36.164′ | 27°33.491′ | 29.27 | 33.75 |  |
| 24 | 2013.8.25 | 122°14.856′ | 29°14.597′ | 28.05 | 32.45 |  |
| 25 | 2013.8.25 | 122°36.056′ | 29°04.962′ | 27.43 | 33.55 |  |
| 26 | 2013.8.25 | 123°18.719′ | 28°40.111′ | 28.78 | 33.36 |  |
| 27 | 2013.8.24 | 123°52.190′ | 28°21.612′ | 28.80 | 33.49 |  |
| 28 | 2013.8.24 | 124°29.626′ | 27°57.670′ | 29.17 | 33.58 |  |
| 29 | 2013.8.24 | 125°03.578′ | 27°38.215′ | 28.87 | 33.56 |  |
| 30 | 2013.8.23 | 125°49.882′ | 27°13.801′ | 28.82 | 34.10 |  |
| 31 | 2013.8.23 | 126°07.246′ | 26°54.124′ | 29.37 | 34.33 |  |
| 32 | 2013.8.25 | 121°46.356′ | 28°12.200′ | 27.72 | 33.34 |  |
| 33 | 2013.8.26 | 122°05.262′ | 28°02.833′ | 27.76 | 33.39 |  |
| 34 | 2013.8.26 | 122°27.773′ | 27°48.286′ | 28.42 | 33.42 |  |
| 35 | 2013.8.26 | 122°54.911′ | 27°34.406′ | 28.68 | 33.22 |  |
| 36 | 2013.8.26 | 123°27.604′ | 27°11.799′ | 28.77 | 33.90 |  |
| 37 | 2013.8.26 | 124°04.012′ | 26°47.120′ | 20.91 | 34.39 |  |
| 38 | 2013.8.28 | 121°09.878′ | 26°32.304′ | 27.92 | 33.51 |  |
| 39 | 2013.8.28 | 121°33.194′ | 26°15.746′ | 28.33 | 33.28 |  |
| 40 | 2013.8.28 | 121°55.176′ | 26°00.185′ | 28.61 | 33.38 |  |
| 41 | 2013.8.27 | 122°36.204′ | 25°32.001′ | 27.25 | 33.64 |  |
| 42 | 2013.8.9 | 122°07.081′ | 32°39.445′ | 25.91 | 30.81 |  |
| 43 | 2013.8.9 | 122°48.299′ | 32°57.379′ | 25.66 | 30.71 |  |
| 44 | 2013.8.9 | 123°07.122′ | 32°57.093′ | 27.44 | 30.62 |  |
| 45 | 2013.8.9 | 123°33.628′ | 33°05.746′ | 28.44 | 30.77 |  |
| 46 | 2013.8.9 | 124°01.808′ | 33°04.324′ | 27.18 | 30.85 |  |
| 47 | 2013.8.8 | 122°17.211′ | 31°59.958′ | 26.58 | 31.03 |  |
| 48 | 2013.8.8 | 122°30.893′ | 32°02.279′ | 24.18 | 32.01 | hypoxic |
| 49 | 2013.8.8 | 123°19.841′ | 32°16.757′ | 26.44 | 31.81 |  |
| 50 | 2013.8.8 | 124°44.211′ | 32°43.964′ | 28.27 | 28.99 |  |
| 51 | 2013.8.15 | 122°13.570′ | 31°00.350′ | 27.30 | 21.55 |  |
| 52 | 2013.8.14 | 121°46.064′ | 31°16.093′ | 30.90 | 0.50 |  |
| 53 | 2013.8.14 | 121°25.600′ | 31°30.232′ | 30.70 | 0.20 |  |
| 54 | 2013.8.14 | 120°56.915′ | 31°46.590′ | 30.50 | 0.20 |  |

**Supplementary Table S2**. Analytical results of ^127^I, ^129^I concentrations and ^129^I/^127^I atomic ratios in the surface water from the East China Sea

| **Sampling station** | **^127^I concentration, (μg/L)** | **^129^I concentration (10^7^atoms/L)** | **^129^I/^127^I atomic ratio**  **(×10^-11^)** |
| --- | --- | --- | --- |
| 1 | 41.04±1.96 | 1.64±0.04 | 8.40±0.23 |
| 2 | 38.67±1.59 | 2.44±0.06 | 13.33±0.36 |
| 3 | 41.76±2.05 | 1.51±0.05 | 7.60±0.25 |
| 4 | 47.18±0.46 | 1.43±0.04 | 6.39±0.20 |
| 5 | 43.39±0.44 | 1.56±0.05 | 7.60±0.21 |
| 6 | 41.67±2.39 | 1.93±0.05 | 9.79±0.27 |
| 7 | 44.35±1.27 | 1.51±0.04 | 7.17±0.21 |
| 8 | 48.43±2.15 | 1.52±0.06 | 6.60±0.26 |
| 9 | 49.78±0.82 | 1.62±0.07 | 6.34±0.25 |
| 10 | 40.91±1.44 | 1.18±0.04 | 6.10±0.21 |
| 11 | 42.52±0.28 | 1.02±0.03 | 5.06±0.15 |
| 12 | 39.67±0.40 | 1.18±0.03 | 6.26±0.18 |
| 13 | 48.43±0.57 | 1.20±0.04 | 5.22±0.18 |
| 14 | 41.13±1.54 | 1.40±0.06 | 7.17±0.29 |
| 15 | 50.15±1.16 | 1.51±0.06 | 6.37±0.20 |
| 16 | 51.87±1.34 | 1.29±0.05 | 5.23±0.16 |
| 17 | 51.51±1.16 | 1.32±0.05 | 5.39±0.18 |
| 18 | 54.74±1.44 | 1.36±0.05 | 5.24±0.16 |
| 19 | 48.05±0.65 | 1.46±0.05 | 6.41±0.24 |
| 20 | 45.99±0.86 | 1.20±0.05 | 6.03±0.23 |
| 21 | 45.38±2.06 | 1.10±0.03 | 4.64±0.14 |
| 22 | 52.05±2.55 | 1.17±0.04 | 4.75±0.14 |
| 23 | 47.55±1.57 | 1.10±0.06 | 4.88±0.25 |
| 24 | 31.37±0.91 | 1.00±0.04 | 6.69±0.25 |
| 25 | 43.74±1.97 | 1.06±0.03 | 5.12±0.15 |
| 26 | 45.82±2.40 | 1.30±0.05 | 6.01±0.22 |
| 27 | 48.00±2.26 | 1.42±0.04 | 6.23±0.18 |
| 28 | 45.16±1.96 | 1.07±0.04 | 5.00±0.19 |
| 29 | 47.33±2.26 | 1.32±0.04 | 5.88±0.19 |
| 30 | 45.24±1.51 | 1.01±0.03 | 4.69±0.15 |
| 31 | 48.46±1.40 | 0.73±0.03 | 3.18±0.12 |
| 32 | 36.97±1.00 | 0.90±0.03 | 5.11±0.19 |
| 33 | 43.31±0.83 | 1.24±0.05 | 6.02±0.21 |
| 34 | 48.03±0.33 | 1.20±0.05 | 5.26±0.21 |
| 35 | 48.16±1.40 | 1.18±0.08 | 5.18±0.30 |
| 36 | 52.59±1.05 | 1.04±0.04 | 4.19±0.15 |
| 37 | 48.47±0.89 | 1.21±0.04 | 5.29±0.13 |
| 38 | 46.76±0.32 | 1.23±0.05 | 5.55±0.22 |
| 39 | 51.87±1.92 | 1.34±0.05 | 5.46±0.18 |
| 40 | 50.29±1.90 | 1.45±0.05 | 6.09±0.17 |
| 41 | 47.94±0.37 | 1.11±0.04 | 4.86±0.18 |
| 42 | 29.87±0.52 | 1.26±0.03 | 8.92±0.25 |
| 43 | 38.82±0.37 | 2.03±0.07 | 11.00±0.36 |
| 44 | 37.57±0.78 | 1.96±0.04 | 11.02±0.23 |
| 45 | 40.12±0.86 | 2.49±0.06 | 13.11±0.30 |
| 46 | 31.29±0.96 | 2.10±0.04 | 14.15±0.29 |
| 47 | 29.80±1.08 | 0.78±0.03 | 5.54±0.22 |
| 48 | 32.65±1.67 | 1.09±0.04 | 7.04±0.27 |
| 49 | 36.68±1.33 | 0.92±0.02 | 5.30±0.13 |
| 50 | 47.18±0.25 | 1.76±0.05 | 7.86±0.24 |
| 51 | 25.03±0.25 | 1.20±0.04 | 10.07±0.37 |
| 52 | 7.07±0.07 | 3.99±0.10 | 119.12±2.99 |
| 53 | 5.00±0.05 | 2.15±0.04 | 90.70±1.84 |
| 54 | 3.06±0.00 | 1.74±0.05 | 101.42±3.34 |

**Supplementary Table S3.** Analytical results of iodide and iodate concentrations and iodide/iodate molar ratios for ^129^I and^127^I in the surface water of the East China Sea

| **Sampling station** | **^127^I^-^ concentration (μg/L)** | **^127^IO_3_^-^ concentration (μg/L)** | **^127^I^-^/^127^IO_3_^-^**(**mol/mol)** | **^129^I^-^ concentration (10^6^atoms/L)** | **^129^IO_3_^-^ concentration (10^6^atoms/L)** | **^129^I^-^/^129^IO_3_^-^**  (**mol/mol)** |
| --- | --- | --- | --- | --- | --- | --- |
| 1 | 35.68 | 5.36 | 6.66 | 8.54 | 7.49 | 1.14 |
| 4 | 26.30 | 20.88 | 1.26 | 10.5 | 3.56 | 2.95 |
| 12 | 5.48 | 27.53 | 0.20 | 5.60 | 5.73 | 0.98 |
| 16 | 14.89 | 38.13 | 0.39 | 3.22 | 9.68 | 0.33 |
| 20 | 10.78 | 34.80 | 0.30 |  |  |  |
| 21 | 9.92 | 35.45 | 0.28 | 3.36 | 6.41 | 0.52 |
| 22 | 20.00 | 33.71 | 0.59 | 0.47 | 0.73 | 0.64 |
| 23 | 10.19 | 37.36 | 0.27 | 3.84 | 6.95 | 0.55 |
| 24 | 11.75 | 19.62 | 0.60 | 1.23 | 8.52 | 0.14 |
| 27 | 7.22 | 40.78 | 0.18 | 4.51 | 9.37 | 0.48 |
| 30 | 10.22 | 35.03 | 0.29 | 2.90 | 6.97 | 0.42 |
| 31 | 6.06 | 42.40 | 0.14 | 1.24 | 5.91 | 0.21 |
| 32 | 7.10 | 29.87 | 0.24 | 3.69 | 5.08 | 0.73 |
| 35 | 14.10 | 42.25 | 0.34 |  |  |  |
| 37 | 18.45 | 39.10 | 0.47 |  |  |  |
| 39 | 12.39 | 40.61 | 0.31 | 4.92 | 8.55 | 0.57 |
| 41 | 2.09 | 47.83 | 0.04 | 2.91 | 7.92 | 0.37 |
| 51 | 24.19 | 0.84 | 28.86 | 5.94 | 5.76 | 1.03 |
| 52 | 4.27 | 2.80 | 1.52 | 23.7 | 15.4 | 1.55 |
| 53 | 1.87 | 3.13 | 0.60 | 21.5 | 1.06 | 20.21 |
| 54 | 2.64 | 0.22 | 11.93 | 17.1 | 1.43 | 11.97 |

Note: The analytical uncertainties (2σ) for ^127^I^-^ in seawater are 5-10% and 2-5% for ^127^IO_3_^-^. The analytical uncertainties for ^129^I are 7-15%, and 5-10% for ^129^IO_3_^-^.

**Supplementary Table S4** Comparison of ^129^I concentrations and ^129^I/^127^I atomic ratios in surface seawater from the ECS and other locations.

| **Coordinate** | **Sampling position** | **Sampling date** | **^129^Iconcentration（×10^7^atoms/l）** | **^129^I/^127^I ratio(×10^-11^)** | **References** |
| --- | --- | --- | --- | --- | --- |
| **Uncontaminated regions** | |  |  |  |  |
| 38-42°N  135E-139°E | Japan Sea | Nov 2007 | 1.3-2.1 | 4.6 -6.9 | [**^13^**](#_ENREF_17) |
| 32-44°N  135-155°E | Western margin of the North Pacific | 2008-2009 | 0.9-1.8 | 2.8-6.2 | [**^14^**](#_ENREF_57) |
| 42°N,146°13E  41°N,138°E;  38°30N,135°E | Pacific Ocean  Japan Sea | Jul-Aug 2007 | --- | (7.1);  (5.8);  (4.6), | [**^15^**](#_ENREF_20) |
| 41°N,141°E  37°N,138°E | Japan Sea | Dec2006;  Feb2007 | 1.6-2.0 | 6.3-8.6 | [**^16^**](#_ENREF_52) |
| 40-75°N  150°E -132°W | North Pacific, Arctic Ocean | Aug–Sep2012; Sep–Oct 2013 | 1.0-14.0 | -- | [**^17^**](#_ENREF_59) |
| 52-72°N  160°E-160°W | Bering and Chukchi Sea | Jun-Oct 1993;  Aug-Sep 1994 | 1.2-3.1 |  | **18** |
| 26.6°N,95°W | Gulf of Mexico | Mar 1992 | 1.7 | 6.3 | **19** |
| 5-36°N  136-173°E | North-western Pacific Ocean; | 1997;1998 | 1.0-3.0 | -- | **20,21** |
| 32–48°S , 51–70°E, | South Indian Ocean | 1997;1998 | 0.9-0.6 | -- | **20,21** |
| 36–63°S  55-68°W | South Atlantic Ocean | Jan 2011 | 0.05-0.4 | 0.3-3.0 | **22** |
| **After Fukushima accident** | |  |  |  |  |
| 35-40°N  140-144°E | Western margin of the North Pacific | May-Oct 2011 | 1.1-89.8 | 4.5–362.3 | [**^14^**](#_ENREF_57) |
| 22 -43°N,  115°E-127°W | Pacific Ocean | May 2011 | --- | 2.2-5.2 | **23** |
| **Contaminated regions** | |  |  |  |  |
| 54-55°N  4-2°W | Irish Sea | 2004-2005 | (3.3-12.8) ×10^4^ | (2.8--8.2) ×10^5^ | **24** |
| 51-55°N  2-6°E | North Sea | Nov 2010 | 921.3-2.4×10^4^ | (0.4-10.2) ×10^4^ | **25** |
| 49-60°N  5-10°E --- | North Sea | 2009 | 280-4.8×10^4^ | --- | **26** |
| 49-58°N  6°W-11°E | English Channel and North Sea | Aug-Sep 2005 | 260-3.6×10^4^ | (0.1-1.7) ×10^4^ | **27** |
| 59-65°N  29-34°E | Baltic Sea | Aug 2009 | (3.2–6.5)×10^4^ | (0.4–1.1)×10^4^ | **28** |
| 54-58°N  14-20°E | Baltic Sea, Kattegat  and Skagerrak | Aug 2006;  Apr 2007 | 250-1.7×10^4^ | (0.4-10.6) ×10^4^ | **29** |
| 54-58°N  8-15°E | Baltic Sea | Nov-1999 | 280-2.3×10^4^ |  | **30** |
| 50-60°N  3-10°E | German Bight;  North Sea;  English Channel;  Irish Sea;  Arctic waters | 2005-2006 | -- | 400-3.0×10^5^ | **31** |
| 54-59°N  2-8°W | Scottish coastal | 2003-2005 | --- | (0.7-33.6) ×10^4^ | **32** |
| 62-89°N  5-155°E | Norwegian Sea; Arctic | July 2001 | 129-3800 |  | **33** |
| 55-59°N  11-13°E | The Swedish west coast | Jul 2007 | 540-5100 |  | **34** |
| 47-50°N  5-8°W | Celtic Sea | Oct–Nov 2010 | 28.7-1170.8 | 122.6-4702.4 | **35** |
| 58-86°N  37°W-25°E | Nordic Seas | May-Jun 2002 | 1–10^3^ | - | **36** |
| 60-62°N,  21-34°W | Iceland and the Irminger Sea | Summer 2010 | 65-337 | 413-1617 | **37** |
| 68-80°N  147°W-30°E | Barents Sea;  Kara Sea;  Western Arctic | 1993 | 58-159;  2.5-69.5 | --- | **38** |
| 47–32°N, 7–15°W | North Atlantic Ocean | Oct-Nov 2010 | 4.0-126.7 | 18.2-569 | **39** |

**Supplementary References**

1. Zhang, J.; Liu, S. M.; Ren, J. L.; Wu, Y.; Zhang, G. L. Nutrient gradients from the eutrophic Changjiang (Yangtze River) Estuary to the oligotrophic Kuroshio waters and re-evaluation of budgets for the East China Sea Shelf. *Prog. Oceanogr.* **74** (4), 449-478 (2007).

2. Beardsley, R. C.; Limeburner, R.; Yu, H.; Cannon, G. A. Discharge of the Changjiang (Yangtze River)into the East China Sea. *Cont. Shelf Res.* **4**(1-2), 57-59(1985).

3. Hsueh, Y.; Schultz, J. R.; Holland, W. R. The Kuroshio flow-through in the East China Sea: A numerical model. *Prog. Oceanogr.* **39** (2), 79-108 (1997).

4. Yang, D. Z.; Yin, B. S.; Liu, Z. L.; Feng, X. R. Numerical study of the ocean circulation on the East China Sea shelf and a Kuroshio bottom branch northeast of Taiwan in summer. *J. Geophy. Res-Oceans* **2011,***116*, 20;DOI10.1029/2010jc006777

5. McCartney, M. S. The subtropical recirculation of Mode Waters. *J. Mar. Res.* **40**, 427-464 (1982).

6. Tsuchiya, M. On the Pacific upper-water circulation. *J. Mar. Res. 40*, 777-799 (1982).

7. Bing-xian, G. Patterns and Structures of the Currents in Bohai, Huanghai and East China Seas. In *Oceanology of China Seas*, Di, Z.; Yuan-Bo, L.; Cheng-Kui, Z., Eds. Springer Netherlands: Dordrecht, 1994; pp 17-26.

8. Lee, H. J.; Chao, S. Y. A climatological description of circulation in and around the East China Sea. *Deep-Sea Res. Part II-Top. Stud. Oceanogr.* **50** (6-7), 1065-1084 (2003).

9. Su, C. C.; Huh, C. A. ^210^Pb,^137^Cs and ^231^Pu,^240^Pu- in East China Sea sediments: sources, pathways and budgets of sediments and radionuclides. *Mar. Geol.* **183**(1-4), 163-178 (2002).

10. Li, W.; Wang, Y.; Wang, J.; Wei, H. Distributions of water masses and hydrographic structures in the Yellow Sea and East China Sea in spring and summer 2011. *Oceanologia et Limnologia Sinica* **43**(3), 615-623 (2012).

11. Nozaki, Y.; Kasemsupaya, V.; Tsubota, H. Mean residence time if the shelf water in the East China and the Yellow seas determined by ^228^RA/^226^RA measurements. *Geophys. Res. Lett.* **16**(11), 1297-1300.12 (1989).

12. Chen, H. Y.; Huh, C. A. ^232^Th-^228^Ra-^228^Th disequilibrium in East China Sea sediments. *J. Environ. Radioact.* **42** (1), 93-100 (1999).

13. Suzuki, T.; Otosaka, S.; Togawa, O. Concentration of iodine-129 in surface seawater at subarctic and subtropical circulations in the Japan Sea. *Nucl. Instrum. & Meth. B* **294**, 563-567 (2013).

14. Suzuki, T.; Otosaka, S.; Kuwabara, J.; Kawamura, H.; Kobayashi, T. Iodine-129 concentration in seawater near Fukushima before and after the accident at the Fukushima Daiichi Nuclear Power Plant. *Biogeosciences* **10** (6), 3839-3847(2013).

15. Suzuki, T.; Minakawa, M.; Amano, H.; Togawa, O. The vertical profiles of iodine-129 in the Pacific Ocean and the Japan Sea before the routine operation of a new nuclear fuel reprocessing plant. *Nucl. Instrum. Meth. B* **268** (7-8), 1229-1231 (2010).

16. Suzuki, T.; Kabuto, S.; Amano, H.; Togawa, O. Measurement of iodine-129 in seawater samples collected from the Japan Sea area using accelerator mass spectrometry: Contribution of nuclear fuel reprocessing plants. *Quat. Geochronol.* **3 (**3), 268-275 (2008).

17. Nagai, H.; Hasegawa, A.; Yamagata, T.; Kumamoto, Y.; Nishino, S.; Matsuzaki, H. Anthropogenic ^129^I in the North Pacific, Bering and Chukchi Seas, and Arctic Ocean in 2012-2013. *Nucl. Instrum. Meth. B* **361**, 680-684 (2015).

18. Cooper, L. W.; Hong, G. H.; Beasley, T. M.; Grebmeier, J. M. Iodine-129 concentrations in marginal seas of the North Pacific and Pacific-influenced waters of the Arctic Ocean. *Mar. Pollut. Bull* **42** (12), 1347-1356 (2001).

19. Schink, D. R.; Santschi, P. H.; Corapcioglu, O.; Sharma, P.; Fehn, U. ^129^I in Gulf of Mexico waters. *Earth and Planetary Science Letters* **135** (1–4), 131-138 (1995).

20. Povinec, P. P.; Lee, S. H.; Kwong, L. L. W.; Oregioni, B.; Jull, A. J. T.; Kieser, W. E.; Morgenstern, U.; Top, Z. Tritium, radiocarbon, ^90^Sr and ^129^I in the Pacific and Indian Oceans. *Nucl. Instrum. Meth. B* **268**, (7-8), 1214-1218 (2010).

21. Povinec, P. P.; Breier, R.; Coppola, L.; Groening, M.; Jeandel, C.; Jull, A. J. T.; Kieser, W. E.; Lee, S. H.; Kwong, L. L. W.; Morgenstern, U.; Park, Y. H.; Top, Z. Tracing of water masses using a multi isotope approach in the southern Indian Ocean. *Earth Planet Sci. Lett***. 302**(1-2), 14-26 (2011).

22. Negri, A. E.; Fernandez Niello, J. O.; Wallner, A.; Arazi, A.; Fifield, L. K.; Tims, S. G. ^129^I Dispersion in Argentina: Concentrations in Fresh and Marine Water and Deposition Fluences in Patagonia. *Environ. Sci. Technol.* **47** (17), 9693-9698 (2013).

23. Tumey, S. J.; Guilderson, T. P.; Brown, T. A.; Broek, T.; Buesseler, K. O. Input of ^129^I into the western Pacific Ocean resulting from the Fukushima nuclear event. *J. Radioanal. Nucl. Chem.* **296**(2), 957-962 (2013).

24.Atarashi-Andoh M, Schnabel C, Cook G, et al. ^129^I/ ^127^I ratios in surface waters of

the English Lake District. *Appl. Geochem***.** **22**(3), 628-636 (2007).

25. He, P.; Aldahan, A.; Possnert, G.; Hou, X. L. Temporal Variation of Iodine Isotopes in the North Sea. *Environ. Sci. Technol.* **48**, (3), 1419-1425 (2014).

26. Christl, M.; Casacuberta, N.; Lachner, J.; Maxeiner, S.; Vockenhuber, C.; Synal, H.-A.; Goroncy, I.; Herrmann, J.; Daraoui, A.; Walther, C.; Michel, R. Status of ^236^U analyses at ETH Zurich and the distribution of ^236^Uand^129^I in the North Sea in 2009. *Nucl. Instrum. Meth. B* **361**, 510-516 (2015).

27. Hou, X. L.; Aldahan, A.; Nielsen, S. P.; Possnert, G.; Nies, H.; Hedfors, J. Speciation of ^129^I and ^127^I in seawater and implications for sources and transport pathways in the North Sea. *Environ. Sci. Technol.* **41**(17), 5993-5999 (2007).

28. Lehto, J.; Raty, T.; Hou, X.; Paatero, J.; Aldahan, A.; Possnert, G.; Flinkman, J.; Kankaanpaa, H. Speciation of ^129^I in sea, lake and rain waters. *Sci. Total Environ.* **419**, 60-67 (2012).

29. Yi, P.; Aldahan, A.; Hansen, V.; Possnert, G.; Hou, X. L. Iodine Isotopes (^129^I and ^127^I) in the Baltic Proper, Kattegat, and Skagerrak Basins. *Environ. Sci. Technol.* **45**(3), 903-909 (2011).

30. Hou, X. L.; Dahlgaard, H.; Nielsen, S. P.; Kucera, J. Level and origin of Iodine-129 in the Baltic Sea. *J. Environ. Radioact.* **61**(3), 331-343 (2002).

31. Michel, R.; Daraoui, A.; Gorny, M.; Jakob, D.; Sachse, R.; Tosch, L.; Nies, H.; Goroncy, I.; Herrmann, J.; Synal, H. A.; Stocker, M.; Alfimov, V. Iodine-129 and iodine-127 in European seawaters and in precipitation from Northern Germany. *Sci. Total Environ***. 419**, 151-169(2012).

32. Schnabel, C.; Olive, V.; Atarashi-Andoh, M.; Dougans, A.; Ellam, R. M.; Freeman, S.; Maden, C.; Stocker, M.; Synal, H.-A.; Wacker, L.; Xu, S. ^129^I/^127^I, ratios in Scottish coastal surface sea water: Geographical and temporal responses to changing emissions. *Appl. Geochem.* **22** (3), 619-627(2007).

33. Alfimov, V.; Aldahan, A.; Possnert, G.; Winsor, P. Anthropogenic iodine-129 in seawater along a transect from the Norwegian coastal current to the North Pole. *Mar. Pollut. Bull* **49**, (11-12), 1097-1104 (2004).

34. Gomez-Guzman, J. M.; Holm, E.; Niagolova, N.; Lopez-Gutierrez, J. M.; Pinto-Gomez, A. R.; Abril, J. A.; Garcia-Leon, M. Influence of releases of ^129^I and ^137^Cs from European reprocessing facilities in Fucus vesiculosus and seawater from the Kattegat and Skagerrak areas. *Chemosphere* **108,** 76-84 (2014).

35. He, P.; Hou, X. L.; Aldahan, A.; Possnert, G., Radioactive ^129^I in surface water of the Celtic Sea. *J. Radioanal. Nucl. Chem***. 299**(1), 249-253(2014).

36. Alfimov, V.; Aldahan, A.; Possnert, G. Water masses and ^129^I distribution in the Nordic Seas. *Nucl. Instrum. Meth. B* **294**, 542-546 (2013).

37. Gomez-Guzman, J. M.; Villa-Alfageme, M.; Le Moigne, F.; Lopez-Gutierrez, J. M.; Garcia-Leon, M. AMS measurements of ^129^I in seawater around Iceland and the Irminger Sea. *Nucl. Instrum. Meth. B* **294,** 547-551(2013).

38. Smith, J. N.; Ellis, K. M.; Kilius, L. R. ^129^I and ^137^Cs tracer measurements in the Arctic Ocean. *Deep-Sea Res. Pt I* **45(6)**, 959-984(1998)

39. He, P.; Hou, X.L.; Aldahan, A.; Possnert, G.; Yi, P. Iodine isotopes species fingerprinting environmental conditions in surface water along the northeastern Atlantic Ocean. *Sci. Rep. 3，(***2013)**. DOI10.1038/srep02685.
